# Newly Identified Deficiencies in the Multiple Sclerosis Central Nervous System and Their Impact on the Remyelination Failure

**DOI:** 10.3390/biomedicines10040815

**Published:** 2022-03-30

**Authors:** Giuseppe Scalabrino

**Affiliations:** Department of Biomedical Sciences for Health, University of Milan, 20133 Milan, Italy; giuseppe.scalabrino@unimi.it

**Keywords:** cobalamin, epidermal growth factor, multiple sclerosis, multiple sclerosis pathogenesis, remyelination failure, normal cellular prions

## Abstract

The pathogenesis of multiple sclerosis (MS) remains enigmatic and controversial. Myelin sheaths in the central nervous system (CNS) insulate axons and allow saltatory nerve conduction. MS brings about the destruction of myelin sheaths and the myelin-producing oligodendrocytes (ODCs). The conundrum of remyelination failure is, therefore, crucial in MS. In this review, the roles of epidermal growth factor (EGF), normal prions, and cobalamin in CNS myelinogenesis are briefly summarized. Thereafter, some findings of other authors and ourselves on MS and MS-like models are recapitulated, because they have shown that: (a) EGF is significantly decreased in the CNS of living or deceased MS patients; (b) its repeated administration to mice in various MS-models prevents demyelination and inflammatory reaction; (c) as was the case for EGF, normal prion levels are decreased in the MS CNS, with a strong correspondence between liquid and tissue levels; and (d) MS cobalamin levels are increased in the cerebrospinal fluid, but decreased in the spinal cord. In fact, no remyelination can occur in MS if these molecules (essential for any form of CNS myelination) are lacking. Lastly, other non-immunological MS abnormalities are reviewed. Together, these results have led to a critical reassessment of MS pathogenesis, partly because EGF has little or no role in immunology.

## 1. Introduction

Traditionally, multiple sclerosis (MS) is considered a chronic acquired demyelinating disease of the central nervous system (CNS) [1,2,3]. Although its cause is still unknown, the pathological hallmarks of MS lesions include demyelinated plaques in the CNS white matter, reactive astrogliosis with glial scar formation, variable degree of axonal and neuronal damage, multifocal neuroinflammation, oligodendrocyte (ODC) loss, leaky blood-brain barrier, and an autoimmune reaction [1,2,3,4]. Furthermore, MS is a heterogeneous disease with respect to its clinical course, response to therapy, neuroimaging, and genetic and histopathological features [1,3,4,5]. Nonetheless, MS is now no longer considered to be a CNS disease limited to CNS white matter demyelination, insofar as the CNS grey matter is affected as well [2,3,4]. MS is a complex disease involving the interplay of CNS resident cells (astrocytes (ASTs), ODCs, neurons (NEUs), and microglia), and immunological-inflammatory cells (T cells, B cells, and macrophages) [3,4,5,6]. We also know that in MS: (a) NEUs are injuried [6,7]; (b) the interaction of one ODC with multiple axons greatly worsens the consequences of the ODC damage; and (c) loss of normal ODC/myelin-axon interaction causes axonal degeneration, which in turn produces not-remitting deficits, thereby leading to severe disability [8,9,10,11]. However, some critical questions about MS remain still unanswered, for instance whether MS is one disease or the manifestation of many diseases, and how genetic factors and/or viral agents and/or environmental factors may contribute to pathogenesis and clinical heterogeneity of MS [8,9,10,11]. From a therapeutic point of view, any specific therapy for MS has to work on cell populations inside the CNS and therefore the molecules must be able to cross the blood-brain barrier.

The epidermal growth factor (EGF) family contains a group of proteins with high structural and functional similarities [12,13]. In addition to EGF, the family members are heparin-binding EGF (HB-EGF), transforming growth factor-α, βcellulin, amphiregulin, and neuregulin (NRG), which all interact with the same ErbB receptor family [14]. In relation to the central nervous system (CNS), EGF was first identified in human cerebrospinal fluid (CSF) [15], and then in the rat brain [16]. Thereafter, the first evidence of the presence of EGF mRNA in the mouse brainstem and striatum has been provided [17]. EGF family members are differently expressed in various CNS parts [18]. The precise physiological roles of EGF and its cognates in the CNS have not yet been defined. The picture is further complicated by the fact that EGF is also produced outside the CNS, mainly by the submaxillary glands, the distal tubules of the kidneys, and the enteric nervous system [19,20,21]. Endogenous and/or exogenous EGF has been shown to penetrate into the CNS through the blood–brain barrier [22,23,24,25]. Another distinguishing feature of the choroid plexus is its transportation of hormones and growth factors into ventricles [26]. Therefore, EGF circulating in the blood might have a direct influence on CNS development and function [24]. In many respects, EGF may be considered as a hormone-like molecule, insofar as it circulates within the blood and can act on far distant cells and/tissues [12].

Vitamin B_12_ (more properly called cobalamin (Cbl)) is a water-soluble vitamin, which contains a single cobalt atom in the centre of a tetrapyrrole ring [27]. The molecule consists of two parts: a planar group (a corrin ring) and a nucleotide set at right angles to each other. The nucleotide consists of a base (5,6-dimethylbenzimidazole) and a phosphorylated sugar, ribose-3-phosphate. The fifth ligand of the cobalt, projecting above the plan of the corrin ring, is bound either to 5′-deoxyadenosyl (generating 5′-deoxyadenosyl-Cbl) or a methyl group (generating methyl-Cbl) [27]. The Cbls are synthesized by many microorganisms, but not by eukaryotic cells. There are only two coenzymatic forms of Cbl, which are involved in two different enzymatic reactions in eukaryotic cells, i.e., mitochondrial methylmalonyl-coenzyme A mutase and cytoplasmic methionine synthase, respectively [27]. Methionine is the precursor of the universal methyl donor S-adenosylmethionine, which is involved in epigenomic regulatory mechanisms. Therefore, the impairment in methionine synthase reaction due to chronic Cbl deficiency leads to epigenomic deregulation, because methylation of DNA, RNA, and histones plays a crucial role in epigenetic and epigenomic mechanisms [27]. Chronic Cbl deficiency significantly correlates with increased micronucleus formation and reduced telomere length [28]. Additionally, we have shown new non-coenzyme Cbl functions in mammalian CNS and elsewhere, because Cbl: a) negatively regulates the synthesis and levels of tumour necrosis factor(TNF)-α and nerve growth factor, and b) positively regulates the synthesis and levels of EGF and interleukin(IL)-6 [27]. Therefore, Cbl is the fulcrum between some myelinotrophins (EGF, IL-6, and p75 neurotrophin receptor) and some myelinotoxins (TNF-α, nerve growth factor, and the soluble CD40:sCD40 Ligand) in the rat CNS. Therefore, when CNS Cbl level is normal, the balance is shifted in favour of myelinotrophins; when CNS Cbl level is abnormally low, the balance is shifted in favour of myelinotoxins. This dysregulation is responsible for the onset and maintenance of Cbl-deficient myelin lesions in mammalian CNS [27]. We have verified this concept in serum and CSF of adult patients with severe clinically confirmed Cbl deficiency [27]. Cbl should be considered an important tessera in the broader mosaic of natural vitamins positively or negatively modulating the synthesis of some cytokines and growth factors in vitro and in vivo in CNS and elsewere [29]. The myelinotrophic action of Cbl has been thoroughly reviewd by many authors [30,31]. On the other hand, different lipid- and water-soluble vitamins other than Cbl are involved in the pathogenesis of some neurodegenerative diseases [32].

The physiological cellular prion glycoprotein (PrP^c^) is anchored to the cell surface through a covalenty attached glycosyl-phosphatidyl-inositol residue and is located at the extracytoplasmatic face of lipid bilayer [33,34]. PrP^c^s are most highly expressed in NEUs, ASTs, ODCs, and microglia, although they are found in many extra-CNS tissues and/organs [35,36,37,38]. PrP^c^s are expressed beginning early in embryogenesis [33,34,35,36]. The immature PrP^c^ undergoes post-translational and proteolytic processing events, generating multiple PrP fragments [35,36,37,38]. After the synthesis in the lumen of the endoplasmic reticulum and the transit through the Golgi apparatus, the fragments and the uncleaved PrP^c^ molecules are trafficked to the cell surface [35,36,37,38]. Given that PrP^c^ molecule lacks an intracellular domain, it may interact with many co-receptors at the cell surface, thus transmitting through downstream signalling pathways [39,40]. To this aim, Akt pathway is also used by PrP^c^s. It is clearly beyond the scope of the present review to recapitulate all the functions of PrP^c^s inside or, even less, outside the CNS. Here, it seems enough to recall that PrP^c^s: (a) play a crucial role in CNS myelin maintenance, although the molecular mechanism(s) through which it occurs, are still elusive [34,35]; (b) bind Cu^++^ hinting at a PrP^c^ role in CNS copper metabolism, although the functional significance of this binding is still to be determined conclusively [35,37,38]; and (c) bind glycosoaminoglycan of the CNS extracellular matrix (ECM) [39,40]. A widespread belief considers PrP^c^s non pathogenic per se, and they ahould be only the basis of their change into a conformationally altered infectious isoform (PrP^sc^). Notwisthanding, we have demonstrated that: (a) an increase in CNS PrP^c^ levels is involved in the pathogenesis of Cbl-deficient central neuropathy [41]; (b) chronic intracerebroventricular PrP^c^ administration to otherwise normal rats reprodude the typical Cbl-deficiency-induced lesions in CNS myelin; (c) repeated intracerebroventricular administration of antibodies (Abs) against the octapeptide-repeat-region of PrP^c^ (a region especially relevant to CNS myelin maintenance [42,43,44]) to Cbl-deficient rats prevented the onset of CNS myelin lesions, without modifying their Cbl-deficient status [41]; and (d) PrP^c^ levels are decreased in CSF and spinal cord (SC) samples taken from patients alive or dead with MS (see further on). Therefore, we introduced the new concept that CNS PrP^c^ levels must be maintained within a well defined range to avoid any CNS myelin damage, and Cbl is one of the physiological molecules doing this [45,46]. It should be noted that the CNS myelinopathies characterized by an increase or decrease in CNS PrP^c^ levels differ from authentic prion diseases in that infectious prions (e.g., PrP^sc^) are not generated, and consequently they could be named “PrP^c^ proteinopathies”, according to Walls et al. [47].

Given that Cbl→EGF→ PrP^c^s are an interconnected sequence of molecules in CNS [48], this review is focused on the effects of EGF, Cbl, and PrP^C^s on the main CNS cells involved directly or indirectly in myelinogenesis, and the derangements of these molecules in MS and MS-like experimental models. This will allow us also to discuss MS pathogenesis on the basis of non-autoimmune criteria, and to try to establish whether the autoimmune and phlogistic reactions in MS are causes or consequences of the disease. Furthermore, the review will discuss some other non-autoimmunity-linked points of MS pathogenesis, in order to tackle the problem from different angles, with an emphasis on some aspects and mechanisms not discussed in a recent review by this author [49].

I think that readers less acquainted with the topic may benefit from some introductory remarks on the CNS myelin structure and its functions, in order to better understand the meaning of “remyelination” in biological terms.

## 2. Basic Concepts Regarding the Biochemistry and Functions of CNS Myelin and the MS Remyelinating Process

CNS myelin is a multilayered stack of uniformly thick and non-conductive plasma membranes, with a characteristic periodic structure of alternating electron-dense and electron-light layers; it is formed in segments, and the unmyelinated axonal areas between segments are called the nodes of Ranvier [50,51,52,53,54]. Given that the different myelin components (i.e., lipids and proteins) are synthesized within ODCs at several subcellular compartments, and are transported, by various mechanisms, to the growing myelin sheaths, CNS myelin is an excellent example of the differentiated products assembled by ODCs to ensure a correct architecture [53,54,55]. Additional to ODCs, other CNS cell types (e.g., NEUs, ASTs, and microglia) are necessary to build the complex structure of CNS myelin [56,57,58,59,60,61,62,63,64,65,66]. The primary function of myelin is to increase the speed of conduction of electric impulses along axons, allowing action potentials to travel long distances at faster rates; in addition, impulse conduction may also be propagated by unmyelinated fibers [50,52]. Myelin sheaths and their subjacent axons must be regarded as functional units that are not only morphologically, but also metabolically coupled [67,68,69]. Precise myelin organization enables the restriction of voltage-gated Na^+^ channels to the nodes of Ranvier, and voltage-gated K^+^ channels to juxtaparanodes [70,71]. The myelinated axons are organized into four domains: the node, paranode, juxtaparanode, and the internode [72]. Myelin is produced mainly postnatally, and is remodelled throughout the lifespan, not only in infancy and adulthood [52,73,74,75,76].

CNS myelin has at least a dual role in providing nutrients to NEUs, and in the energy conservation of axons through the saltatory conduction of impulses [68,77]. It is also widely known that a virtuous circle exists between axons and CNS myelin sheaths, insofar as the former strongly contribute towards the development and integrity of ODCs and myelin maintenance, and the latter provide trophic and metabolic support to the former [68,74,78]. It follows that CNS demyelination in MS induces complex morphological and ultrastructural damages, the blockage of impulse conduction, axonal loss, NEU loss, disorganization of Ranvier’s nodes, and ion channel segregation [76,79,80,81]. The degeneration of demyelinated axons is traditionally considered to be the major cause of the permanent neurological disability eventually endured by MS patients [82,83,84,85]. It should also be noted that the death of a single ODC results in myelin loss, and in the interruption of electrical conduction in many axons, because a single ODC myelinates portions of multiple adjacent axons [67]. Conversely, timely remyelination in MS protects denuded axons rather than newly generated ODCs [84,85,86,87]. The myelin diameters vary much less than during CNS developmental myelination [88,89,90,91,92]. MS remyelinated areas are characterized by myelin of paler color, the so-called “shadow plaques” [92,93,94,95,96,97]. The discrepancy between new and old myelin is not surprising, because the replacement of a lost part of a tissue and/or organ in human beings frequently involves some biochemical and/or morphological differences between the original and the replaced tissue. For instance, this has been observed in: (a) so-called “liver regeneration”, in which the synthesis of some fetal proteins (e.g., α-1-fetoprotein) reappears, and the typical organization of lobules is often lost [98,99]; and (b) the left ventricular hypertrophy, in which the intercalated discs show ultrastructural changes associated with sarcomere assembly/disassembly, and fetal β-myosin heavy chains are re-expressed [100,101]. Similarly, the remyelination process is associated with the re-expression of developmental genes by ODCs [102].

Efficient MS remyelination entails at least six steps: (a) the differentiation of neural stem cells (NSCs) towards the neuroglial lineage; (b) NSC migration towards the lesion(s); (c) the proliferation of oligodendrocyte precursors (OPCs); (d) OPC migration towards the demyelinated axons; (e) OPC differentiation towards ODCs, and their interactions with the demyelinated axons; and (f) ODC myelination and wrapping [103,104]. Therefore, the process of any type of myelination by ODCs entails a stepwise progression from precursor specification to differentiated ODCs. This process is also coordinated by a series of transcriptional and chromatin remodelling events [104,105,106,107]. Furthermore, it should be noted that numerous factors have been identified that control the differentiation and proliferation of OPCs, and the myelination by ODCs [104,105,106,107]. These include extracellular factors as well as ODC-intrinsic transcription factors, epigenetic modulators (e.g., DNA methylation, “non-coding” RNAs) (see further on), and signaling pathways [90,106,108,109]. In more detail, the crosstalk between transcription factors and epigenetic modulators is surely a regulatory point in the OPC → ODC developmental processes [110,111,112,113]. OPCs can transduce extracellular signals to transcription factors, which recruit protein complexes containing activators or repressors, allowing for the activation or repression of genes regulating ODC lineage determination, proliferation, migration, and eventually myelination [113].

It is widely recognized that remyelination occurring in MS (a) differs greatly in relation to different CNS areas, and the status of disease progression; (b) is limited and variable in patient populations; (c) is more robust in early phases rather than in late phases of the disease [92,96,114,115]; and (d) OPCs proceed through the same stages of maturation towards myelinating ODCs during remyelination (however induced) [81,104]. Therefore, the CNS remyelination occurring after demyelinating diseases has been considered as incomplete, rather than delayed myelination by most authors [58,88,89]. Nonetheless, MS remyelinated areas are more vulnerable than the normal appearing withe matter areas to new demyelination [87,116]. The developmental mechanisms reused by the adult CNS during myelin remodeling, or changes after myelin injury (however induced), are far from clear.

## 3. The Role of EGF in the Genesis and Maintenance of CNS Myelin

It is clear that EGF is only a tessera of the intricate mosaic of CNS myelination. Herein, the effects of EGF on NSCs, ODCs, and ASTs will be briefly summarized, as they have been thoroughly reviewed in a recent publication by this author [49]. Unfortunately, studies on the effects of EGF on most of the other tesserae of the mosaic are still lacking.

EGF increases the proliferation, as well as the growth, of multilineage neurospheres [117,118]. It is worth noting here that neurospheres (i.e., clonal aggregates of NSCs) are induced by EGF in the fetal, embryonic, and adult striatum and hippocampus of humans, rats, and mice, which clearly indicates that, in these organisms, the CNS is capable of generating different OPCs and/or ODCs, and other CNS cell types, regardless of its differentiation and age [119,120,121,122,123,124,125,126,127]; this needs to be considered when assessing the potential of the CNS to recover during the course of MS or experimental MS-like models [128,129]. In the postnatal rat cerebral cortex and in the subcortical white matter of the adult human brain, a population of multipotent cells generates mature glial progeny and NEUs, and is highly responsive to EGF, in terms of cell proliferation, because it has EGF receptors [130,131,132].

The crucial effect of EGF on the proliferation and differentiation of NSCs was discovered in the 1990s [117,133]. NSCs are distributed throughout different regions of the mammalian CNS (e.g., the subventricular zone (SVZ), the subgranular zone of the dentate gyrus, the subcallosal zone, and the spinal cord (SC)) [134], and act as a reservoir of multipotent cells; furthermore, they can be expanded, using suitable stimuli, during and after CNS demyelinating diseases induced in experimental animals by various means [135,136]. The most prominent difference between SC NSCs and those located within the brain is that the former are mostly quiescent during normal conditions, with low proliferative activity; however, they become highly proliferative upon SC injury, e.g., in experimental allergic encephalomyelitis (EAE) [137]. NSCs contribute to the generation of new ODCs more than parenchymal OPCs, at least in the CNS of mice fed cuprizone, inducing CNS demyelination [127]. NSCs are capable of self-renewal, which prevents their pool from becoming depleted. This would result in the partial inability of OPCs to generate new, mature ODCs under normal conditions and/or after a demyelinating injury [138]. Indeed, many OPCs differentiate and develop into ODCs, although a significant amount remain as OPCs into adulthood [139].

NSCs play a major role in relation to CNS demyelinating insults, as they can be committed to a neuronal or glial cell lineage by external factors (e.g., EGF and/or other neurotrophic growth factors [140,141,142]); therefore, NSCs affect the recovery capacity of the CNS in MS, and after chemically- and/or virally-induced demyelination [143,144]. NSCs play a key role not only from a regenerative point of view, but also because they multipotently develop ODCs, ASTs, and NEUs, i.e., three CNS cell types, the integration of which is one requirement for the occurrence of physiological CNS myelination [129,145]; in addition, small quantities of Schwann cells are partially derived from the peripheral nervous system, and are involved in CNS remyelination [129,146]. NSCs can be mobilized in vivo from the SVZ to demyelinated CNS areas [123,147,148,149,150,151,152]. Moreover, after demyelinating CNS insults, OPCs located close to the demyelinated areas migrate and undergo terminal differentiation to replace lost ODCs [144]. In keeping with this, proliferating ODCs have been discovered to be present in both active and chronic inactive MS plaques [153]. Together, these findings demonstrate that: (a) the adult CNS is endowed with OPC regenerative capacity; (b) not all OPCs differentiate in ODCs,and therefore, a population of OPCs can persist throughout the lifespan and retain the ability to proliferate and differentiate into ODCs; and (c) the MS CNS is capable of recruiting new ODCs [153].

It is well known that ODCs become mature, myelinating cells in response to a combination of different molecules, with opposing stimulatory or inhibitory effects (e.g., growth factors, hormones, neurotransmitters, components of ECM, chemokines, and some transcription factors) [92,103,105,154,155]. ODCs take on their typical biochemical patterns, including myelin assembling, myelin basic protein (MBP), proteolipid protein (PLP), myelin-associated glycoprotein (MAG), and myelin ODC glycoprotein (MOG) [53,92]. PLP and MBP reside within compacted myelin, and represent the two most abundant proteins of myelin itself [57,156]. In more detail, MBP is involved in the adhesion of the cytosolic surfaces of myelin by binding to negatively charged lipids, as well as in the binding of actin filaments to the surface of lipid vesicles [156]. The ODC genes encoding myelin-associated proteins, i.e., MAG, MBP, and PLP, are strongly induced during myelination [157]. PLP is essential for axonal function, MBP for myelin compaction and wrapping, and MAG for myelination initiation [56,74,158].

It is noteworthy that white matter OPCs are prone to proliferation, and contribute to adult oligodendrogenesis, whereas gray matter OPCs are mostly quiescent, or slowly proliferative, and may therefore remain in an immature state [103].

The well documented effects of EGF on ODCs and OPCs include promoting the in vitro induction of SVZ ASTs to differentiate into migratory OPCs and ODCs [159,160], whereas anti-EGF antibodies (Abs) greatly reduce in vitro OPC migration, and silence EGF receptor activation [161]. EGF is, therefore, one of the most powerful and multifarious epigenetic chemical messengers determining the fate and maturation of NSCs, ASTs, and the OPC→ODC lineage, although other myelin- and/or ODC-trophic growth factors, such as platelet-derived growth factor (PDGF), insulin-like growth factor (IGF), fibroblast growth factor, brain-derived neurotrophic factor, transforming growth factor-β, and ciliary neurotrophic factor, play a role in the differentiation and proliferation of the different CNS cell lineages [57,87,91,162,163,164,165,166,167].

ODCs from neonatal rat cerebral cortex, and from the adult human temporal lobe, contain NRGs [168,169] and express the ErbB receptors for NRGs [169], hinting at the possibility of autocrine and/or paracrine signaling. This suggests that adult human ODCs may play a role in regulating their own development, as well as influencing the development of surrounding glial cells. Although the proliferation and differentiation of ODC lineage maturation are mutually exclusive processes, some factors, including EGF, have been shown to promote the proliferation, and subsequent differentiation, of OPC→ODC lineage cells [170]. Additionally, there is the transcriptional regulation of ODC lineage maturation, showing a complex regulatory system [57,62,108,110,157]. The crosstalk between extrinsic signals (e.g., growth factors and molecules of the CNS ECM) and intrinsic factors (e.g., transcription factors and chromatin modifiers of ODCs) leads to a balance between repressive signals that maintain the OPC status and prevent its differentiation, and de-repressive signals that support ODC differentiation and myelination [171] (see below). The expression level of relevant transcription factors during the differentiation and maturation of ODCs is one of the most important factors in the regulation of this cell lineage and in CNS myelination, insofar as high expression of inhibitors of differentiation and myelination is present in OPCs [157]. All of these inhibitors are rapidly downregulated at the pre-myelinating and myelinating stages, while some pro-differentiation transcription factors concomitantly appear [157].

ASTs secrete most of these myelin- and/or ODC-trophic factors [172], and most of the ECM components [92]; they can also function as mediators of CNS myelination by promoting OPC migration, proliferation, and differentiation [61,63,92,104,173]. Furthermore, ASTs control the availability of CNS iron, which is a key factor for the survival and proliferation of ODCs, and is stored as non-heme iron in ODCs and myelin [174]. ASTs also support remyelination by supplying lipids to ODCs [91]. ASTs secrete HB-EGF and highly express EGFR, hinting at an autocrine function of ASTs via HB-EGF [175]. It has been reported that: (a) a gradual decrease in HB-EGF expression occurs as ASTs mature; and (b) conversely, a promotion of the immature state of ASTs coincides with increasing HB-EGF [175]. This is one example of the ways through which ASTs support myelination [61,176].

EGF has also been shown to induce the activity of glutamine synthetase (GS; EC 6.3.1.2), a phenotypic marker of ASTs [177,178]. The EGF induction of GS merits particular attention because ASTs are key cells in the maintenance of glutamatergic neurotransmission [179]. The glutamate released by glutamatergic NEUs is taken up by ASTs through specific transporters, which convert it into glutamine by means of GS; the glutamine released by ASTs is taken up by NEUs, and eventually reconverted into glutamate by means of phosphate-activated glutaminase [179]. GS expression has also been found in ODCs (mostly confined to the white matter), but not in OPCs [180,181,182,183], thus challenging the concept that CNS GS is exclusively expressed in ASTs and, therefore, the two-compartment model (NEU-AST) of the glutamate–glutamine metabolic cycle [182]. Intriguingly, mice lacking ODC GS do not show any impairment in CNS myelination [182]. However, it has been shown that glutamate regulates the proliferation of OPCs as well as their differentiation into myelinating ODCs [184], and induces myelin formation after its release by axons [185]. This makes glutamate signaling a powerful mechanism that allows NEUs and ASTs to regulate the development of OPCs. After AST ablation, CNS myelin maintenance is hampered by the elevation of local glutamate levels, and demyelination has been observed in mouse SC [59]. All of these findings emphasize the key role of GS in NEU–AST–ODC crosstalk [183,185,186]; in addition, GS has been shown to be involved in ammonia detoxification, which is key to preventing hyperammonemia-associated neurotoxicity.

EGF directly stimulates IGF synthesis by ASTs [187,188], which is involved in the positive regulation of OPC differentiation [146]. IGF modulates MBP levels in cultured neonatal rat OPCs [189], is indispensable for mature ODC survival [190], induces the differentiation of multipotent adult neuronal progenitor cells into ODCs [191], and modulates OPC Akt activity during the terminal phase of differentiation [155].

NEUs positively influence OPC mitosis and promote oligodendrogenesis by means of their electrical activity [64,72,75,185,192,193]. NEUs also synthesize NRGs, i.e., some EGF-like factors regulating OPC proliferation and differentiation [194]. Moreover, communication between NEU-oligodendroglia is mediated through glutamate and γ-aminobutyric acid [186].

EGF stimulates the CNS synthesis of PrP^C^s, which are also produced by ODCs [48], and are widely acknowledged to be involved in the generation and maintenance of CNS myelin (see also above) [48]. EGF is also a positive regulator of thyroid cell proliferation [195]. Triiodothyronine plays a key role in the timing of OPC differentiation by stopping OPC proliferation and starting their terminal differentiation [196,197,198,199,200,201,202,203,204]. Consequently, triiodothyronine has been successfully used to treat EAE [199,205], cuprizone-induced CNS demyelination [200,206,207], and MS [208]. Nevertheless, the picture is further complicated by the fact that several classes of hormones, other than triiodothyronine, regulate oligodendrocytogenesis, and are able to induce the maturation of, and myelin production by, ODCs [203,204,209]. IGF has also been shown to reduce demyelination in a rat EAE model [210,211].

**Figure 1:**
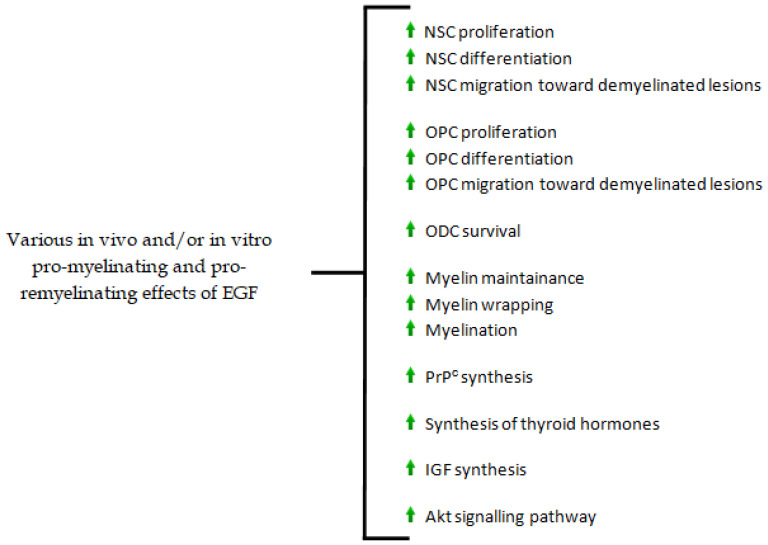
The main effects of epidermal growth factor (EGF) on oligodendrocytes (ODCs) and astrocytes (ASTs). The green arrows indicate stimulation. See text for details and references. IGF = insulin-like growth factor; NSC = neural stem cell; OPC = oligodendrocyte precursor cell; PrP^C^ = normal cellular prion protein.

The main EGF effects on CNS cells, CNS myelin, and various mechanisms involved in CNS myelinogenesis, are shown in Figure 1. (The green arrows indicate stimulation).

**Figure 2:**
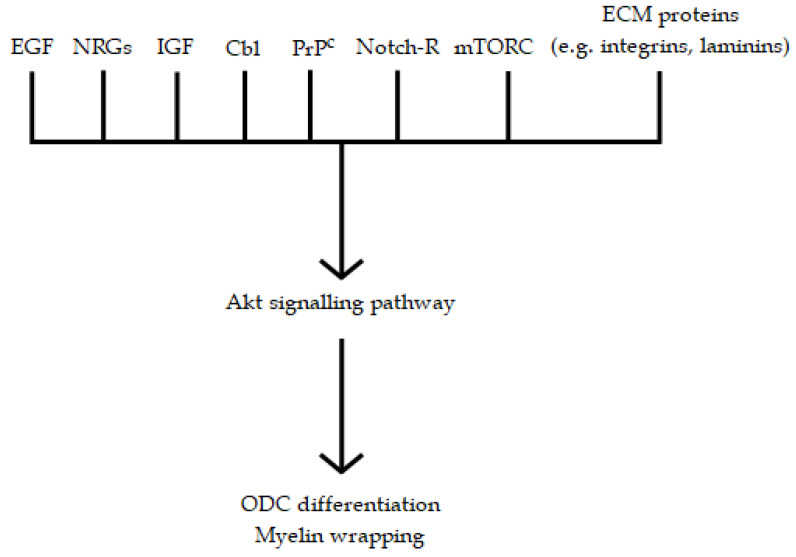
Various physiological molecules of the central nervous system (CNS) act through the Akt signaling pathway. The pivotal role of the Akt signaling pathway in CNS myelination is evident. See text for details and references. Cbl = cobalamin; ECM = extracellular matrix; EGF = epidermal growth factor; IGF = insulin-like growth factor; mTORC = mammalian target of rapamycin complex; NRG = neuregulin; ODC = oligodendrocyte; PrP^C^ = normal cellular prion protein.

It is interesting to note that the serine/threonine kinase Akt is a common step in the signaling pathways of EGF [212,213], Cbl [214], IGF-1[215,216], Notch [217], NRGs [218], NF-κB transcription factor [219], and the mammalian target of rapamycin [57,220] (see Figure 2).

It is also worth noting that transgenic mice with increased ODC Akt expression show hypermyelination [221], emphasizing the pivotal role of Akt in CNS myelinogenesis [222,223]. The other two principal pathways regulating the proliferation and differentiation of, and myelination by, ODCs are the extracellular signal-related kinase 1 and 2 (Erk 1 and Erk 2)/mitogen-activated protein kinase pathway [215,223,224,225,226], and the wingless-related integration site (Wnt)/intracellular β-catenin signaling cascade (which negatively regulates OPC differentiation, myelination, and remyelination) [226,227,228,229]; however, conflicting results have been reported [230,231]. A Wnt/βcatenin increase in MS tissues has been reported [182,215,228,229].

Notch signaling is expressed in OPCs, ODCs, and ASTs [232,233,234], and its intracellular domains are induced by, and crosstalk with, EGF [235]. Notch1 has been shown to inhibit in vitro OPC differentiation and myelin formation [236]. Notch regulates some of the ODC transcription factors that influence the myelin gene expression of ODCs [237]. EGF can also modulate some transcription factors, e.g., those in the Id family (e.g., Id2 and Id4 which inhibit OPC differentiation [108,164]), in the opposite way (reviewed in [49]). Furthermore, AST-derived endothelin-1 inhibits OPC differentiation and remyelination, through ODC Notch activation and Jagged induction, in reactive ASTs of lysolecithin-induced demyelination in the mouse CNS [238], but it enhances in vitro migration of rat OPCs [239]. Importantly, the EGF–Notch interaction also occurs in the SVZ cells during normal developmental myelination and/or remyelination, and regulates the size of the local OPC pool [209].

The pro-inflammatory transcription factor, nuclear factor kappa-light-chain-enhancer of activated B cells (NF-kB) pathway (canonical/classical and non-canonical/alternative) deserves a special attention, because it is widely known to be involved in the maintenance of myelin normal status rather than in myelin formation of the CNS [240,241]. The NF-kB proteins are constitutively active at high levels in NEUs and are tightly regulated by interactions with the inhibitor of kB (IkB) family [242]. Broadly summarized, many studies have shown that NF-kB is activated in the MS brain [242,243,244,245], noticeably in ODCs of active lesions [246]. Nonetheless, it is exceedingly difficult to establish how much the increase is due to inflammatory/immunological cells present in MS lesions [247] and how much it is due to neuroglial cells, including ASTs. ASTs play surely an important role in MS pathogenesis and remyelination in MS, although it is still to be defined [248,249,250,251,252,253,254]. In the past, we have contributed to solve. albeit indirectly, this problem, insofar as we demonstrated that: a) the levels of activated NF-kB (particularly the p50 and p65 subunits) are increased in rat SC during chronic Cbl deficiency (i.e., a purely myelinolytic CNS disease without any histopathological feature of inflammation and/or immunological reaction [27,255]); and b) the NF-kB increase is TNF-α-mediated [256]. Therefore, it is conceivable that a down-regulation of CNS NF-kB levels are required for a normal CNS myelin maintenance. As for ASTs in MS, although their beneficial or detrimental role is still controversial, it is worth noting that it has been shown that activated ASTs produce HB-EGF [257], which in turn blocks NF-kB activation in intestinal epithelial cells [258].

The CNS myelin status depends on the balance between myelin-building and myelin-destroying factors located within ODCs and the ECM [56,154,164,170,202,216,218,259,260,261,262,263,264,265,266,267,268,269,270,271]. The intrinsic factors and extrinsic factors that impose oligodendrogenic effects on NSCs and OPCs have been thoroughly reviewed [167,176]. Furthermore, physiological and repair myelination, and myelin maintenance in the CNS, require the correct expression and combination of various CNS growth factors and membrane-bound cell molecules, particularly integrins [272,273,274]. EGF increases NSC membrane levels of the β_1_-integrin,which is involved in axonal–glial interactions [275], Akt-dependent myelin wrapping [276], crosstalk with the Notch pathway [277], and interactions with several growth factors that regulate the number and development of ODCs in time and space [273], and control axonal ensheathment by ODCs [273,276]. It is worth noting that: (a) ODCs variably express α and β subunits of integrin receptors based on developmental stage [278]; (b) ODC differentiation and maturation is also impacted by integrin signaling; (c) ODC integrin receptors also modulate signaling via several growth factors (e.g., NRG, PDGF, and EGF) [278]; and (d) integrins interact with some ECM molecules [278]. The CNS tetrad including integrins, growth factors, ECM molecules, and growth factor receptors, plays a pivotal role in influencing both myelination and remyelination through an integrated network of processes concerning the differentiation and proliferation of ODCs [278].

The primary aim after severe CNS myelin insult and/or diseases (including MS) is to develop and reinforce the CNS regenerative capacity before resorting to stem cell transplantation. Mobilizing NSCs for CNS remyelination is a rather unexplored perspective in MS therapy [167,279], and EGF seems to be able to perform this task (see above). It is also clear that the basal approach to any successful remyelinating therapy is to identify ways of enhancing the endogenous remyelination process based on the precise knowledge of why the remyelination process fails in MS. In other words, active therapeutic interventions that promote OPC maturation and/or prevent ODC destruction seem to be critical for allowing efficient remyelination and functional recovery of axons [97]. Once again, EGF seems to be a promising candidate for this task, although by no means unique.

Nature has provided the human species with an available reservoir of progenitor cell lines in the normal adult CNS (i.e., NSCs and OPCs) [77,80,167,176]. At first glance, this statement could seem to be inappropriate from a scientific point of view, and even a teleological one. Instead, it simply reports what is, in reality, present in the mammalian CNS, and may represent a strong chance for exploring a successful therapy of MS. Indeed, it is worth noting that: (a) NSCs are abundant and present throughout the CNS, multipotently develop ODCs, ASTs, and NEUs (see above), and, in response to demyelination, are activated and generate OPCs, which migrate to the CNS demyelinated lesions [143,150]; (b) subsequently, these OPCs undergo ODC lineage steps, produce the great majority of remyelinating ODCs [129,145], and remain proliferative throughout the lifespan, even in the adult CNS, in response to local demyelination [155]; (c) adult OPCs have a transcriptome that is more similar to that of ODCs than that of neonatal OPCs [280]; (d) OPCs can give rise to small quantities of remyelinating CNS Schwann cells, which are only partially derived from the peripheral nervous system [129,146]; and (e) mature ODCs are still capable of dividing [281].

Taking advantage of the endogenous reservoir of neuroglial progenitors (i.e., NSCs) and enhancing their ability to migrate, differentiate, mature, and eventually myelinate, seems to be the most appropriate course of action, not least because we do not know of any physiological molecule(s) that can be used to inactivate remyelination inhibitors [167]. It is known that the main organs (e.g., heart, liver, kidneys, and lungs) have a functional reserve capable of enhancing their “work” within maximal performance limits, and this increased performance is achieved through different cellular mechanisms that vary from one organ to another. Why should the CNS lack this functional “residual” capacity? The four main sources of ODCs that contribute to the remyelination process after demyelination are as follows: (a) SVZ-derived NSCs (see above); (b) local OPCs; (c) mature surviving ODCs that reside either in, or adjacent to, demyelinated areas; and (d) Schwann cells [79,97]. In other words, all the aforementioned findings provide a crucial proof-of-principle for directing the NSC→ODC lineage steps into a successful path of endogenous remyelination, if suitably stimulated by EGF and/or other OPC/ODC-trophic growth factors [58,282], without leaving chromatin remodelling and transcriptional factors out of consideration [157]. This notion should also be emphasized because the infusion of autologous bone marrow-derived mesenchymal stromal cells in MS patients has been shown to be an ineffective therapy [283], although conflicting results have been reported [284,285,286].

Various inhibitors of oligodendroglia differentiation are physiologically present in CNS ECM, and are of paramount importance in accounting for MS remyelination failure [80,103,105,154,155,263]. This topic has been reviewed by various authors [260,287,288], although no effect of EGF on these physiological inhibitors of normal ECM has been investigated so far.

## 4. CNS EGF Changes in MS and Experimental MS-like Models

MS is widely considered to be the paradigm of human CNS demyelinating diseases, despite the fact that (a) the gray matter of the brain and SC are also affected by demyelination [289,290,291,292,293,294,295]; (b) gray matter lesions differ from those of white matter in terms of the number of activated ASTs and microglia [292]; (c) atrophy is more marked in gray than in white matter [289]; (d) gray matter damage becomes increasingly dominant as MS progresses [290]; (e) SC gray matter demyelination is significantly greater than white matter demyelination [296,297]; (f) the extent of remyelination in cortical lesions is consistently more extensive [298,299,300,301]; and (g) intracortical lesions are not associated with increased lymphocyte infiltration when compared with typical MS lesions and the cortices of control patients [302]. Various findings claim to explain these differences between gray and white matter in the extent of demyelination and/or remyelination, such as the following: (a) the qualitative and/or quantitative differences between gray matter OPCs and white matter OPCs [299]; (b) ODC lineage cells continuously produce myelinating ODCs in white matter, whereas the majority of ODC lineage cells of gray matter remain in an immature state; (c) the different expression of the ECM molecules inhibiting OPC differentiation, which may be higher in white matter ASTs than in gray matter ASTs [91,301]; (d) adult white matter ASTs are less supportive of in vitro myelination than gray matter ASTs [300]; (e) a greater proportion of OPCs present in remyelinated cortical lesions than in remyelinated white matter lesions [299]; and (f) a difference in the comparative lipid profiling of gray matter from that of white matter, the former being enriched in polyunsaturated fatty-acid-containing phospholipids, the latter being enriched in sphingolipids, such as cerebrosides [303]. Ultimately, however, remyelination fails in both gray and white matter, contributing to severe disease progression [300,301,304].

The various experimental MS-like models (immunologically-induced EAE, chemically- or virally-induced CNS demyelination, and transgenic animals) do not entirely reproduce the histopathological features, clinical course, and/or CSF abnormalities typical of MS, but only mirror some of its characteristics [209,305,306,307,308,309,310], because of the heterogeneity of MS lesions, and the complexity of the repair mechanisms in MS [92,97,311]. Furthermore, the effectiveness of these experimental models in the development and testing of new drugs for the MS treatment has proved to be limited, and this has raised substantial doubts as to whether the experimental models and human diseases have the same pathogenesis [312]. Of course, the toxin-induced CNS demyelination models in rodents lack the autoimmune component of human MS [209]. There is another reason to think that experimental MS-like models poorly reflect the MS situation, i.e., the difference in ODC generation dynamics and adaptive myelination between humans and rodents [97,171,313]. The different animal models of experimental MS-like demyelination have been reviewed [79,80,209,314].

Even from an immunological point of view [315,316,317], EAE models differ from MS because (a) the experimental autoimmunity is preferentially mediated by CD4^+^ T cells [318], whereas MS patients generally have more CD8^+^ than CD4^+^ T cells [1,319,320], which initiate the lesions, whilst the former amplify the lesions [316]; (b) anti-MOG Abs have rarely been observed in MS patients [321,322,323]; (c) B cells play a dominant role in MS [324,325], but do not contribute to CNS damage in most EAE models [312]; (d) the absence or paucity of lymphocytic infiltration has been observed in acute MS lesions [326,327]; and (e) anti-TNF -α Abs greatly improve the course of EAE in animal models, but have been shown to worsen the clinical course of MS [306,312]. Furthermore, it is exceedingly difficult to gauge how informative the rodent models of remyelination are for MS remyelination, because: (a) the kinetics of ODC generation is very different between rodents and humans in healthy condition; and (b) the neuropathology is different in humans and the experimental models used in animals [313].

Generally speaking, it can be said that the demyelination process in chemically induced CNS demyelination is temporally separated from the remyelination process, whereas both processes occur concurrently in EAE, and that chemical models of CNS demyelination are primarily used to study remyelination, whereas the EAE induced by MOG or MBP is mainly used to assess the immune component of the disease [328,329]. However, mice in which EAE has been induced by a fusion of the protein of MBP and proteolipid protein (MP4) have Abs against NEUs, and the glia of their enteric nervous system and histopathological gastrointestinal lesions are similar to those observed in the enteric nervous system of MS patients [330].

**Figure 3:**
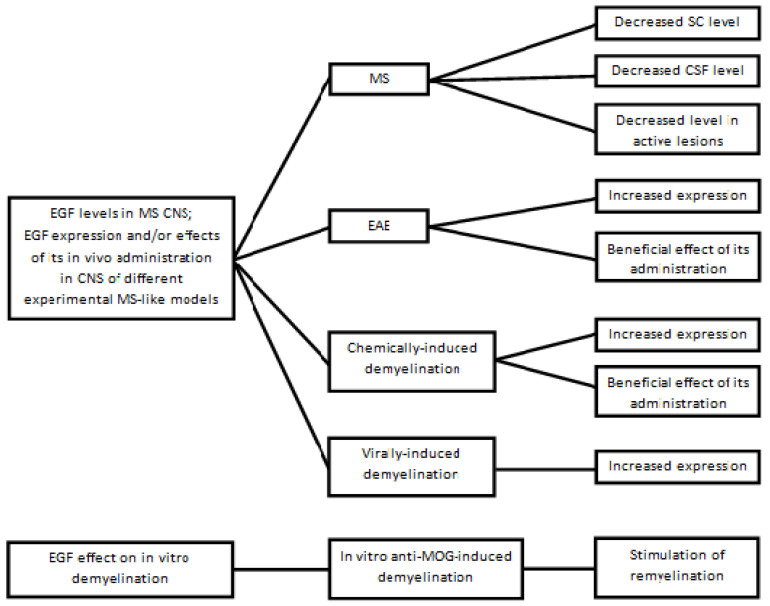
Schematic diagram of the epidermal growth factor (EGF) levels in multiple sclerosis (MS) central nervous system (CNS), EGF expression, and the effects of its in vivo or in vitro administration in different models of experimental allergic encephalomyelitis (EAE) and chemically or virally-induces CNS demyelination. See the text for details and references. CSF = cerebrospinal fluid; MPG = myelin oligodendrocyte-specific glycoprotein.

EGF and other myelin- and/or ODC-trophic growth factors, such as IGF, and PDGF, are overexpressed in rodents with experimental MS-like demyelinating diseases [88,331,332,333,334,335,336], but the expression of myelinotrophic growth factors during and/or after experimental CNS demyelination (however induced) differs from that observed during CNS developmental myelination [81,90,104]. Furthermore, a cocktail of the many different neurotrophic growth factors, other than EGF, that promote the differentiation and proliferation of OPCs and ODCs, has been effective in stimulating corpus callosum remyelination in cuprizone-fed mice [337]. The lack of EGF in the MS CNS, as well as the positive effects of EGF administration into rodents in different MS-like models, have been reported and discussed exhaustively in a recent review by this author [49]. Figure 3 shows (a) the main changes in CNS EGF expression in some models of adult CNS demyelination in vivo and in vitro; (b) the in vivo and in vitro effects of EGF administration in some experimental MS-like models; (c) CNS EGF levels in MS patients (also recently reviewed in [49]); and (d) EGF increase during the remyelination process of aggregating fetal rat brain cells treated in vitro with anti-MOG antibody to induce MS-like demyelination [338]. These cultures do not model all aspects of myelination effectively [339].

Given that there is a large body of evidence indicating the positive involvement of EGF in CNS myelinogenesis and myelin maintenance, it is conceivable that the EGF deficiency that we found in the MS CNS is causally linked to MS remyelination failure, although this is only one factor responsible for it. Surprisingly, there is a report showing that local NRG administration does not improve remyelination in rats in which CNS demyelination is induced by the gliotoxin ethidium bromide [340].

From a theoretical point of view, it is tempting to make a comparison, albeit cautiously, between the pathogenesis of MS and that of Parkinson’s disease, although the two diseases differ considerably. The levels of dopamine in the striatum of Parkinson’s patients progressively decrease over the years because of NEU loss from the substantia nigra [341], therefore dopamine replacement is the mainstay of treatment; however, although this therapy improves the neurological symptoms and lengthens the survival of patients, it does not substantially change the unfavourable prognosis. By analogy, it is conceivable that that EGF progressively decreases in the MS CNS (at least during some clinical courses). On the basis of the results of our studies on EGF in MS CNS, we posited that in vivo EGF administration should be therapeutically effective in an EAE model [342]. It is worth highlighting that EGF administration has been shown to be effective in inducing remyelination in two other models of EAE, other than the one we used (see again Figure 3).

The studies of EGF levels and/or in vivo EGF administration in experimental MS-linked models (noticeably EAE) support the view that the autoimmune reaction in the SC white matter of EAE mice may actually be caused by damage to, or abnormalities in, the structure of CNS myelin and/or by ODC pathology, rather than the other way round, as is traditionally believed, because EGF does not seem to have any immunological function identified so far [343]. It is conceivable that the beneficial EGF effects may be also due to EGF-induced triiodothyronine secretion (see above) and/or EGF-induced prolactin levels, in agreement with MS remission during pregnancy [209,344,345,346]. Among the growth factors studied to date, EGF is the only one growth factor whose levels have been investigated both in CSF and SC samples of MS patients [49]. The abnormally low EGF levels we found in the MS CNS support the notion that this is involved, at least in part, in the failure of remyelination, and this could also be relevant for the scarce support of the surviving ODCs in MS lesions [301].

The development of any in vivo treatment that prevents CNS injury, or repairs the axonal–glial interface, is a lofty aim, but one that is necessarily doomed to failure if the cause of the disease is unknown. The repair of CNS local demyelination necessarily involves local OPC recruitment through their proliferation and migration. Then, OPCs to engage and demyelinate axons and differentiate into remyelinating ODCs (see above). Therefore, the factors whose absence accounts for remyelination failure, should be identified, as well as the factors whose presence accounts for the inhibition of endogenous remyelination. A “therapeutic” strategy to promote endogenous CNS remyelination using certain growth factors, which are effective in OPC maturation and ODC myelination, has been proposed by different authors, and it was often successful in the experimental MS-like models (reviewed in [49,58,209,282,335]). At present, no remyelinating therapies focused on enhancing endogenous factors are clinically available. Moreover, the efficiency of some myelinotrophic growth factors in attenuating EAE “clinical” and/or histopathological features does not necessarily translate into a successful MS therapy strategy. Evidence of this has been obtained by the failed clinical trials with IGF-1 in MS patients [58], even though IGF modulates the immune system by inducing lymphocyte proliferation [347,348]. IGF administration to mice with EAE provided only mild protection when given before disease onset, but did not modify the disease course when given after disease onset [332]. Unlike EGF, CSF IGF levels are not different in MS patients when compared to controls [282].

Myelinotrophic EGF effects have been shown in experimental CNS-damaging models other than those that are MS-linked. For instance, intranasal HB-EGF administration immediately after chronic neonatal hypoxia decreases ODC death and enhances the proportion of ODCs from OPCs in the mouse CNS [349], and EGF promotes the in vitro recovery and regrowth of the injured, as well as uninjured, processes of ODCs [350]. Furthermore, CNS EGF deficiency seems to be not specific for MS, because a similar decrease has been found also in patients with Parkinson’s disease [351].

Although it is hazardous to infer that the results obtained in rodents can be translated directly to humans and vice versa, EGF seems to be a theoretical candidate for MS therapy, probably together with an immunosuppressant drug. As a matter of fact, EGF has been shown to (a) expand and to mobilize the SVZ progenitor pools after different types of CNS myelin damage; (b) generate new ODCs from these progenitors; and (c) inhibit signaling pathways (e.g., Notch) that arrest OPC maturation and proliferation [209] (see above). Nevertheless, the level and/or expression of EGF in the mouse CNS soon after the onset of EAE, or toxin-induced demyelination, have not been investigated so far.

## 5. Cbl in MS CNS

Here, it would be enough to recall some points relevant to the topic of the review, which are as follows: (a) no ultrastructural evidence of new myelin deposition occurring simultaneously with myelin damage has ever been found in the CNS of adult Cbl-deficient rats or humans [27,31,255,352,353]; (b) no changes in the main classes of neurolipids have been found in the SC of Cbl-deficient rats, unlike in the MS CNS [27]; (c) the lesions of Cbl-deficient CNS are purely myelinolytic [27,255,352,353]; (d) EGF-related mRNA synthesis ceases in the SC of Cbl-deficient rats, together with their CSF EGF levels, although both of them are restored after Cbl replacement therapy [27]; (e) in vivo EGF administration has been shown to be as effective as Cbl in “curing” the CNS myelinolytic lesions of Cbl-deficient rats without modifying their Cbl-deficient status [27]; and (f) repeated intracerebroventricular administration of anti-EGF Abs to otherwise normal rats brings about SC myelinolytic lesions similar to those in the SC of Cbl-deficient rats [27]. Therefore, EGF has to be considered the physiological effector of the Cbl myelinotrophic effect.

The debate concerning the possible role of Cbl in MS has been long-lasting, and the results are conflicting (reviewed in [354,355,356]). Nevertheless, it must be emphasized that phlogosis, demyelination, axonal damage, immune reaction, and astrocytic scars (i.e., typical features of MS) have never been observed in human Cbl-deficient central neuropathy (i.e., subacute combined degeneration) or in the Cbl-deficient CNS of different animal species [27,255,352,353].

We found that CSF Cbl levels significantly increased in relapsing–remitting (RR) and secondary-progressive (SP) patients in comparison with the controls, but the increase observed in the primary-progressive (PP) patients did not reach the level of statistical significance [357]. This finding clearly indicates the loss of the positive Cbl-mediated regulation of CSF EGF levels in the RR and SP patients. The total homocysteine (tHCYS) levels in the MS SCs were also statistically significantly lower than those in the control SCs [357]. The simultaneous decrease in the Cbl and tHCYS levels of MS SC is paradoxical, because it is widely known that Cbl deficiency increases tHCYS levels due to impaired methionine synthase activity (EC 2.1.1.13) in all of the mammalian tissues so far investigated [27,31](see also Introduction). To the best of my knowledge, this is the first report of a tissue in which decreased Cbl levels are associated with decreased tHCYS levels. Although the pathophysiological significance of Cbl increase in MS CSF remains a matter of speculation, it is difficult to interpret it as a mirror of increased Cbl availability in MS CNS. Instead, it is conceivable that the increase in CSF Cbl levels may be due to either the only partial recruitment of Cbl into MS CNS cells, or to abnormalities in the blood–brain and/or blood–SC barriers in MS [358]. Although these hypotheses are not mutually exclusive, the decreased Cbl levels in the MS SC we found [359] seem to be in keeping with the former hypothesis. Moreover, we can reasonably exclude that this dichotomy between CSF Cbl levels and those of SC is due to reduced Cbl transport in MS CSF, as there were no changes in CSF levels of holotranscobalamin (which binds biologically active Cbl) in any of our MS patients, regardless of their clinical course, in comparison with the controls [359]. Furthermore, it is unlikely that the lack of Cbl in the MS SC is responsible for any aberrant regulation of gene expression and/or epigenetic changes occurring in remyelinating ODCs [111,360,361,362,363,364,365], because DNA hypomethylation of the white matter from MS brains is due to increased DNA demethylation activity rather than a decrease in methyltransferase activity [361,362,364,365]. The dichotomy we observed between high CSF Cbl levels and low SC Cbl levels supports the notion that a “therapeutic” Cbl administration during the MS course seems to be useless, because of an apparently reduced permeability of the blood–spinal cord barrier to Cbl. However, a previous study reported that combination therapy with interferon-β plus Cbl improved both “clinical” and histopathological pictures of mice with EAE [366].

Recently, it has been shown that Cbl can regulate glial migration and synapse formation [367]. Furthermore, ASTs that express megalin (a receptor binding various complexes, including the transcobalamin–Cbl complex, and necessary for Cbl absorption [368,369,370]) can bind Sonic hedgehog (Shh), which is subsequently released to induce the proliferation and migration of OPCs and ODCs by regulating the expression of ODC transcription factors, e.g., *Olig1* and *Olig2* (with a predominant role of the latter) [106,197,371]. Anti-megalin Abs completely blocked OPC proliferation [371]. Nonetheless, some ODCs are able to differentiate and proliferate via a Shh-independent mechanism [372]. Total Shh has been found to be decreased in the cortical white and gray matter of post-mortem MS samples in comparison with controls [373].

EAE autoimmunity is preferentially mediated by CD4^+^ T cells, whereas MS patients often have more CD8^+^ T cells than CD4^+^ T cells (see above) [1,319,320,374,375]. As it has been shown that Cbl is an immunomodulator that regulates the ratio of CD4^+^/CD8^+^ T cells and natural killer cell activity in humans [376,377,378], we should theoretically consider the possibility that Cbl deficiency in the MS SC may also play a role in determining the local immunological response. It has been shown that Cbl deficiency causes a decrease in the number of CD8^+^ cells [376,377,378], which, however, are clonally expanded in MS lesions [379,380]. Therefore, the lack of Cbl in the MS CNS seems to impinge only on myelin damage and remyelination failure; therefore, the possibility that SC Cbl deficiency may be a contributory factor in the immunological abnormalities of MS remains merely speculative.

## 6. Role off PrP^C^s in CNS Myelin and MS SC

PrP^C^ has two isoforms, one of which is membrane-bound, the other is soluble (=secreted) [381]. In NEUs, PrP^C^s are transported along axons [382]. Important evidence of the role of PrP^C^s in myelin maintenance comes from studies of PrP^C^-KO mice and/or mice lacking one or more parts of the PrP^C^ molecule [383]. Although PrP^C^-KO mice display no overt neural phenotype, a variety of subtle phenotypes (including those with SC myelin damage) have been reported in these mouse strains [384]. Furthermore, some strains of transgenic mice overexpressing some parts of the PrP^C^ molecule, or expressing *PRNP* gene mutants with the deletion of different PrP^C^ regions, show severe myelin lesions of the SC and/or brain [385,386,387]. CNS PrP^C^s have, therefore, the fundamental task of maintaining myelin integrity [34,35], although they are not alone in doing this. This role is also supported by PrP^C^ binding to many CNS molecules involved in myelin maintenance, such as certain ECM proteins (e.g., laminins and glycosaminoglycans) and transmembrane proteins (e.g., neural cell adhesion molecule and integrins) [35,381]. Furthermore, increased MBP citrullination, which has been well characterized in MS and has been shown to correlate with the severity of CNS demyelination in a transgenic mouse model [388], has frequently been observed in CNS diseases other than MS, e.g., in the prionopathy Creutzfeldt–Jakob disease [389]. It is worth noting that PrP^C^ absence in isolated cortical OPCs enhances proliferation, but decreases differentiation in the mouse CNS [390].

As mentioned in the Introduction, there is a growing body of evidence suggesting that abnormalities in the CNS PrP^C^ levels can be involved in the pathogenesis of some neurological diseases that are different from the group of neurodegenerative diseases, commonly called prionopathies (reviewed in [45,48]). Indeed, the local opposed deregulation (excess or deficiency) of the synthesis and/or levels of PrP^C^s in the CNS jeopardize normal myelin maintenance without any conformational change in PrP^C^ molecule, as in the case of scrapie prions (PrP^sc^). This notion is also supported by our findings, which show that: (a) increased CNS PrP^C^ levels play a key role in the myelin damage brought about by Cbl deficiency in the rat CNS [45]; (b) CSF PrP^c^ levels have been found to be significantly increased in patients with severe clinically confirmed Cbl deficiency; (c) the PrP^C^ levels in the post-mortem MS SC samples are significantly lower than those in the control SC, without any substantial difference in relation to the degree of demyelination of the post-mortem SC samples used in the study [359]; and (d) the CSF PrP^C^ levels in the patients with RR-MS and PP-MS were both significantly lower than those in the controls, but they were unchanged in patients with amyotrophic lateral sclerosis or Alzheimer’s disease [45]. PrP^C^s have been previously described as neuroprotective [34,391], but PrP^C^ overexpression has been associated with increased neurotoxicity, myelin damage, and cell death [44,45,386,387]. This means that CNS PrP^C^s should be constrained to a certain level in order to develop their natural functions. Moreover, the Notch-1 transcription factor is activated in the NEUs of animal and human brains with prionopathies [392].

At first glance, Cbl, EGF, and PrP^C^s seem to have nothing in common. However, if we look more closely, we can see that they have a number of similarities. First of all, most PrP^C^-related and Cbl deficiency-related diseases seriously damage the myelin of the CNS and peripheral nervous system [27,393]. Second, acquired Cbl-deficient central neuropathy, and most prionopathies, have a long latency period before manifesting themselves clinically [27,34,35,48]. Third, we demonstrated, for the first time, that Cbl and EGF buffering of SC PrP^C^ levels is crucial for maintained SC myelin at normal levels, because normal CNS levels of Cbl and EGF protect SC myelin against the myelin-damaging excess or lowering of SC PrP^C^s levels [48]. Fourth, SC is the CNS part most severely affected by chronic acquired Cbl deficiency, and in MS [46,231]. In many respects, however, these diseases differ considerably, for example: (a) CNS reactive astrocytosis and microglial activation have been observed in these diseases, but it is still unclear whether reactive astrocytosis plays a beneficial or detrimental role (or both) in MS myelin lesions and remyelination [27,63,248,249,250,251,252,253,254]; (b) ODCs are certainly affected in MS, and are involved in its pathogenesis, but not in prionopathies, and very rarely in Cbl-deficient central neuropathy (at least from an ultrastructural point of view) [46,48]; (c) spongy vacuolation (i.e., intramyelinic and interstitial edema) is a typical feature of the CNS white matter in Cbl-deficient central neuropathy, and is very often observable in the CNS gray matter of humans and/or animals with prionopathies [45,48]; and (d) unlike MS, prionopathies and chronic Cbl-deficient neuropathies (both acquired and inherited) do not show any histological and/or ultrastructural signs of CNS remyelination [48,389].

EGF has been shown to increase PrP^C^ gene expression in cultured mouse ASTs [394]. Our in vivo studies demonstrated that EGF stimulates the PrP^C^-related mRNA in the SC of Cbl-deficient rats and, together with Cbl, contributes to maintaining SC and CSF PrP^C^ levels within the normal range [48]. The typical response of Cbl-deficient SC to Cbl replacement consists of increased PrP^C^-mRNA levels (and the consequent maintenance of local normal PrP^C^ levels) concomitantly with the reappearance of ultrastructural normal SC myelin [48]. EGF therefore plays a dual role in CNS myelin maintenance, i.e., as the only known effector of Cbl myelinotrophism and myelin maintenance in CNS, but also, per se, as the stimulator of PrP^C^ synthesis in the mammalian CNS [48].

On the whole, the Cbl→EGF→PrP^C^s sequence is of paramount importance in maintaining myelin architecture and function, and the chain of events emanating from any derangement in this sequence leads to the heavy damage of CNS myelin.

Last but not least, SC PrP^C^ deficiency in MS may (a) exacerbate the autoimmune reaction, as has been shown on the CNS of mice with EAE but with a normal immune system [395,396,397]; and (b) contribute to remyelination failure, because PrP^C^s are not only fundamental molecules for CNS myelination [48], but also their absence from the CNS delays ODC differentiation in the mouse CNS [390].

## 7. The Horns of the Dilemma of MS Pathogenesis: “To Be or Not to Be an Autoimmune Disease—That Is the Question”

In MS, axonal degeneration has been extensively studied, but what triggers it has remained obscure [70,82]. In other words, autoimmunity, inflammation, and demyelination in MS represent a three-cornered hat, but it is still unclear what is the starting point of the disease. The key point is to understand whether the autoimmune attacks to ODCs are a cause or consequence of the disease. Therefore, it is very important to identify what triggers the autoimmune and phlogistic reactions in MS, although it is unlikely that only one CNS abnormality can explain the pathogenesis of the disease and the failure of remyelination [5,261,329,398,399,400].

The aporias of the autoimmune pathogenesis of multiple sclerosis have been thoroughly discussed in a number of reviews [82,326,401,402,403,404,405]. There is no proof-of-principle that MS is an autoimmune disease, and the immune response to myelin antigens has not been associated with the onset and/or progression of MS [401]. The traditional view of MS pathogenesis highlights the role of CNS myelin loss, as it leads to the impaired propagation of action potentials across the areas of demyelinated axons, and it is the major cause of neurological disability. Therefore, the search for mechanisms that primarily cause the demyelination of white matter has always been the focus of research [406].

Past research focused on the autoimmune-mediated pathogenesis of the disease has led to the development of anti-MS-demyelination drugs, which can be roughly grouped into two main sets, i.e., immunosuppressant drugs and anti-remyelination inhibitors. Nonetheless, current anti-MS treatments merely relieve the neurological symptoms. MS patients treated with appropriate immunosuppressive therapy still show increasing clinical disability and disease progression, as a result of remyelination failure [328,407,408,409,410].

Some main findings supporting a non-autoimmune-mediated pathogenesis of MS (e.g., abnormalities in the CNS myelin structure and in the OPC→ODC lineage maturation, CNS glutamate excitotoxicity, abnormal CNS levels of glial fibrillary acidic protein (GFAP), inhibitory molecules in the CNS ECM, and abnormal levels of CNS PDGF and EGF) have been previously discussed in a recent review by this author [288]. Further findings dealing with a non-autoimmune pathogenesis of MS, will be discussed here in in terms of abnormalities in mitochondria, microRNAs (miRNAs), neurosteroids, and epigenetic changes in the MS CNS.

### 7.1. Mitochondrial Abnormalities in MS

Several studies have demonstrated mitochondrial respiratory chain deficiency and a direct link between mitochondrial dysfunction and ODC myelination [86,411,412]. Other mitochondrial dysfunctions described so far are: (a) the progressive accumulation of mitochondrial DNA mutations, likely related to deficient mitochondrial DNA repair; and (b) the increased production of reactive oxygen species. Therefore, there is compelling evidence to suggest an important role of mitochondrial dysfunctions in MS pathophysiology, because mitochondrial defects in demyelinated axons can be a factor determining their higher vulnerability [411,412]. Nonetheless, there is indirect evidence that the axonal mitochondrial damage by demyelination may be an intrinsic reaction to myelin destruction [413,414,415]. Reduced mitochondrial gene expression (particularly in the transcripts of the electron transport chain) was found to be specific to the neurodegeneration of the MS cortex [416].

A significant decrease in mitochondrial content in remyelinated axons when compared with demyelinated ones has been reported [413,414,415]. Therefore, the mitochondrial contents in remyelinated axons do not return to the levels in non-demyelinated and/or myelinated axons of MS and controls [86], as has been observed in the myelin ultrastructure of remyelinated CNS areas (see above). However, a conflicting report shows that the mitochondrial content within remyelinated axons in remyelinated areas do not significantly differ from that of demyelinated axons [417]. Furthermore, multiple deletions of mitochondrial DNA have been found throughout the gray matter in MS CNS, leading to respiratory chain deficiency within NEUs [363,413].

### 7.2. Neurosteroid Abnormalities

In addition to the adrenals and the gonads, steroidogenesis is also present in the CNS, insofar as NEUs and glial cells synthesize steroids (e.g., pregnenolone and progesterone) that regulate neural function through autocrine and/or paracrine mechanisms [418]. These CNS steroids (termed “neurosteroids”) are active in parallel to, but independent of, endocrine signaling by extra-CNS peripheral steroids [418]. The active form of vitamin D (called calcitriol) functions as a steroid hormone acting via both genomic and non-genomic pathways [418]. The term “neurosteroids” is misleading in many respects because they are not a new class of steroids, but are classical androstane- and pregnane-derived molecules. Indeed, neurosteroids are different from peripheral steroid hormones as several neurosteroids have no identified nuclear hormone receptor, and they generally act directly upon plasma membrane ion channels [419]. Of course, the identification of a given steroid in the CNS does not provide direct information on its site of production [420]. NEUs, ODCs, and other glial cells have been identified as sites of neurosteroid synthesis [419]. Neurosteroids can stimulate myelination and/or remyelination directly via ODCs, or indirectly via NEUs and ASTs, because reciprocal communication between these three types of CNS cells is mandatory to synthesize CNS myelin [72] (see above). It is worth highlighting that the activity of 5α-reductase is significantly higher in mature ODCs compared with OPCs, leading to the increased formation of allopregnanolone precursor 5α-dihydroprogesterone [419]. As a result, treatment with progesterone or allopregnanolone has been shown to be effective in the remyelination treatment of EAE and in chemically-induced CNS demyelination, in part because progesterone promotes the differentiation of OPCs into myelinating ODCs [421,422].

Neurosteroids and peripheral steroids play a critical role in CNS myelinogenesis, as has been demonstrated by defective remyelination when progesterone biosynthesis was stopped [419]. Strong, indirect evidence in favour of a hormonal influence on MS clinical course is the well-known sex differences in the incidence of the disease, and by the pregnancy-associated changes in the disease relapse [209,422].

Levels of neurosteroids, particularly allopregnanolone and dehydroepiandrosterone, have been found to be very low in MS brain white matter [421]. These alterations in CNS steroidogenic machinery may be micro(mi)RNA-mediated [421]. Neurosteroids also may regulate the inflammatory “côté” of MS [423], and EAE models reflect the inflammatory reaction occurring in RR-MS, although this does not necessarily reflect the true cause of the disease [424]. Of note, however, chronic EGF treatment in mice with MOG-induced EAE has been shown to be much more effective than the dexamethasone treatment in terms of improvement in histopathological SC features (including inflammation) and the “clinical” course [288]. It is almost redundant to mention here that glucocorticoids (even intrathecally [425]) are often used in MS therapy [426], mainly because of their well-known immunosuppressive effects at pharmacological doses [347]. Furthermore, glucocorticoids suppress the production of NF-κB [347], as Cbl does in rat SC [256]. Furthermore, transgenic mice with selectively inactivated astroglial NF-κB showed reduced myelin damage after SC injury [427], and improved outcome in EAE [428,429]; further still, selective inhibition of NF-κB strongly reduced CNS demyelination after cuprizone feeding in mice [430]. Together, these results pinpoint the pivotal role of the CNS NF-κB level for maintaining normal CNS myelin levels, regardless of the type of CNS disease [27,431]. It is, therefore, conceivable that the downregulation of CNS NF-κB levels is required to preserve CNS myelin compaction, because (a) Cbl-deficient central neuropathy, which is not an inflammatory disease of the CNS and affects mainly ASTs [298], shows high NF-κB levels in the SC of Cbl-deficient rats [256]; (b) transgenic mice with selectively inactivated astroglial NF-κB showed reduced myelin damage after SC injury [427], and improved outcome in EAE [428,429]; and (c) selective inhibition of NF-κB strongly reduced CNS demyelination after cuprizone feeding in mice [430].

A dysfunction of neurosteroid synthesis leading to a widespread CNS dysmyelination has been suggested as the first key factor in MS pathogenesis, triggering a local abnormal immune response [432]. In keeping with this hypothesis, a study showed that neurosteroidogenesis (e.g., dehydroepiandrosterone) is strongly reduced in MS ODCs, together with decreased levels of the enzymes synthesizing the hormone [433].

### 7.3. MS Epigenetic Abnormalities in OPC→ODC Cell Lineage

Here, only some epigenetic abnormalities of MS, not previously discussed in a review by this author [287], will be mentioned. The main epigenetic mechanisms involve histone modifications and variants, DNA methylation, ATP-dependent chromatin remodelling, and non-coding RNAs [107,111,434,435]. It is becoming increasingly clear that the loss of some epigenetic mechanisms regulating gene expression in the OPC→ODC lineage contributes to the pathogenesis of MS [361,362,363,436]. Furthermore, it should be important to characterize the cell types showing aberrant epigenetic profiles in MS, and correlate the findings to specific neuropathological features [361,362,436]. Efficient DNA methylation is necessary for remyelination in the SC of mice with lysolecithin-induced CNS demyelination [363,437,438].

Higher levels of histone acetylation in ODCs of chronic MS lesions have been found in comparison with the controls [107,439], whereas ODCs of early MS lesions express deacetylated histones [107,435]. Given that histone acetylation impairs ODC differentiation and remyelination, these findings could explain, at least in part, the remyelination failure in MS [361]. These changes are associated with the high expression of transcriptional inhibitors of ODC differentiation [439].

Decreased methylation in CpG islands in the white matter of MS brains has been found to be the result of higher DNA demethylase activity than that in normal brains [361], and to be specific for MS [362].

#### miRNA Abnormalities

miRNAs, a group of several thousand small, single-stranded, “non-coding” RNAs, which are normally composed of 20–25 nucleotides, are derived from larger hairpin-folded RNA precursors; they are present in both the gray and white matter of the CNS, and influence approximately one-third of all protein-coding proteins [440,441,442]. miRNAs influence protein expression post-transcriptionally by means of the inhibition of translation or the degradation of targeted messenger RNA transcripts [441]. miRNAs can leave the intracellular environment where they normally function, and enter the blood and CSF. The expression of miRNAs is controlled by genetic and epigenetic mechanisms; for instance, by DNA methylation and/or specific histone modifications [443]. miRNAs bind to the 3′-untranslated region of their target mRNA, although the 3′-untranslated region of one target mRNA may contain several sites where different miRNAs may bind [440,441,442]. In other words, a single miRNA can regulate hundreds of transcripts, thus having a broad array of functional consequences.

miRNAs are involved in neurogenesis, ODC differentiation, and myelin formation [62,444]. ODC-specific miRNAs either increase the ODC number and myelination, or repress ODC gene expression, which maintains OPCs in their proliferative, non-differentiative state [445]. Therefore, ODC lineage maturation is controlled at transcriptional (see above) and post-transcriptional levels [62]. Transgenic mice, in which miRNAs have been disrupted in OPCs and ODCs, show abnormal development of CNS myelination, because some miRNAs of the ODC lineage enable the rapid transition from proliferating OPCs to myelinating ODCs [446,447]. Indeed, some miRNAs (e.g., miRNA-219 and miRNA-338) are specifically involved in the regulation of myelination, and in myelin maintenance of the CNS [447], and have been found to be expressed at reduced levels in demyelinated MS lesions [448]. On the contrary, the miRNA-125 family has been found to be upregulated in MS active lesions, and in OPCs isolated from SC of EAE mice, but not in OPCs isolated from the spontaneously remyelinating corpus callosum of mice with lysolecithin-induced CNS demyelination [449]. In keeping with this, the CSF levels of the same miRNA family are significantly elevated in MS patients compared to healthy controls [450]. These findings support the notion that oligodendrogenesis is impaired when miRNA-125 is overexpressed, whereas remyelination is accelerated when miRNA-125 is downregulated. miRNA profiles in MS CSF are dysregulated in comparison with those of patients with other neurological diseases [451,452].

CNS miRNAs have been found to be differentially expressed between MS subtypes and the normal CNS [444,453,454,455]. It is therefore difficult to describe the typical profile of MS miRNAs, not least because these alterations may derive from CNS cells and/or the immune cells that have infiltrated into the MS CNS parenchyma [441,444,453,455]. Furthermore, it is worth underlining that nearly fifty miRNAs are upregulated, and nearly thirty are downregulated, in MS [440,444,455,456,457]. Therefore, it is exceedingly difficult to say whether the abnormal miRNA expression in MS CNS contributes towards triggering the initial phase of the disease, and there are relatively few studies of miRNA profiles in MS CNS [441]. Recently, several miRNAs (i.e., miR-146 and miR-219) have been shown to promote remyelination in the corpus callosum of mice fed on a cuprizone diet [458,459,460].

Unfortunately, to date, there are no studies investigating the effect(s) of different myelinotrophic growth factors on miRNA profiles during demyelination and/or remyelination in the CNS. For further information concerning the relationships between miRNAs abnormalities and MS, readers are referred to a recent review [455]. The role of ODC-specific, long non-coding RNAs in MS remains to be investigated [436].

## 8. Conclusions: “Ad Impossibilia Nemo Tenetur” (“Nobody Is Compelled to Do What Is Impossible”)

The pathogenesis of MS has always posed a real puzzle for neurologists and neuropathologists, insofar as the key point to be clarified is whether CNS myelin damage occurs as a consequence of autoimmune and/or inflammatory processes, or whether, vice versa, it occurs starting from myelin itself and/or the myelin-producing ODCs, and leading subsequently to autoimmune and/or phlogistic processes. In other words, this crucial puzzle represents the proverbial “chicken and egg” conundrum, and it highlights the complexity and the interdependence of demyelination and inflammatory/autoimmune processes in the MS CNS [96,461,462].

Since Rudolf Virchow coined the word “myelin” in 1864 [76,463,464], we have made enormous strides in our understanding of myelin structure and composition, biosynthesis of CNS myelin, ODC biology, and myelination steps by ODC themselves. Therefore, we can highlight some pivotal points in the puzzle of MS pathogenesis, such as: (a) EAE models do not directly resemble MS because they only recapitulate some manifestations of the disease; (b) toxin-induced CNS demyelinating models provide advances in investigating the cellular mechanisms of demyelination and remyelination; (c) the transgenic mouse models of MS have been mainly constructed on the basis that MS could be a purely autoimmune disease [461,465,466], thus shedding little light on our understanding of MS pathogenesis; (d) on the whole, the different animal models of MS have been useful for testing new drugs for the MS therapy [467,468,469]; and (e) the studies of blood abnormalities in MS patients have been of limited use, if any, in our understanding of MS pathogenesis because it is indisputable that MS is a CNS disease, and, therefore, we must look for its aetiology inside the CNS itself, not elsewhere. In this regard, it should be stated that the examination of the MS CSF has been widely considered as highly fruitful in MS research [470,471,472]; however, the number of brain banks around the world is still too little, and their number should be enhanced.

If the restrictive preconception of the autoimmune-mediated pathogenesis of MS were to be abolished, and non-autoimmune-mediated pathogenesis were to receive the respect it now so clearly deserves, only time will tell us how fast and how far it will carry our understanding of MS pathogenesis. The autoimmune-mediated pathogenesis of MS seems now to be increasingly fading. Indeed, much of the attention has derived from some findings in MS patients, that are reported in Table 1.

These findings seem to be in disagreement with, or at least independent of, the autoimmune-mediated pathogenesis of MS. From an epistemological point of view, the theory of the autoimmune pathogenesis of MS can be exposed to the possibility of “falsification”, thus satisfying Popper’s criterion [473]. Indeed, Karl Popper introduced the falsificationist methodology to science, which requires that any scientific theory and/or hypothesis must be disproved [473]. Therefore, the demarcation criterion between theory and experience is not the verification, but the falsifiability.

The controversy around an autoimmune-mediated or a non-autoimmune-mediated pathogenesis of MS is long-lasting, and it is strengthened by the absence of an appropriate equivalent animal model (see above) and the heterogeneity of the disease [96,399,400]. The presence of autoimmune and phlogistic reactions is undeniable, but the possibility cannot be discounted that an alternative pathogenesis of MS may exist and possess a still unknown trigger, likely associated with abnormalities in myelin structure and/or ODCs and/or the CNS levels of some myelinotrophic growth factor(s) (notably EGF and PDGF). It is important that mice with EAE and treated with NRG showed CNS lesions with more remyelination than in controls, and this correlated with increased expression of MBP exon 2, a marker for remyelination [474], in agreement with our results [342].

The non-immunological findings listed in Table 1 lead us to suppose that autoimmune and inflammatory reactions in MS should be considered as the consequence, rather than the cause, of the disease, because most of the findings have been obtained from samples from patients with MS. Nonetheless, it is well established that the immune system directly participates in the destruction of myelin and, eventually, NEUs.

The lingering debate on the autoimmunity as the primary cause of MS has been debated from an epistemological point of view in a recent review by this author [288]. Here, some key facts underpinning a non-autoimmune pathogenesis of MS should be recapitulated: (a) the lack of two pivotal myelinotrophic growth factors (namely EGF and PDGF) in MS CNS [282,288,335], although a dysregulation of other growth factors can not be ruled out [475]; (b) abnormalities in the differentiation, maturation, and transcriptome profile of MS ODCs [157,197,475,476]; (c) different epigenetic abnormalities, mainly in ODCs [157]; (d) identification of many myelin inhibitors in ECM [262,477,478]; (e) in vivo EGF administration has been shown to be effective in the remyelination of different MS-like models (see Figure 3); and (f) EGF is lacking in CSF and SC taken from MS patients (see above). Therefore, further studies are needed that investigate non-immunological molecules at the onset of the disease.

It is well known that Giovanni Battista Morgagni, the founder of modern pathology, correlated the histopathological lesions observed post-mortem in the organs to the symptoms of the patients. In his book “De sedibus et causis morborum per anatomen indagatis” (“Of the sites and causes of diseases as investigated through anatomy”) (Venice, 1761), the word “morbus” (disease) was used to describe symptoms and their cause(s), which were thought to be localized by identifying the morphological and histopathological alterations in the affected organ(s). From a methodological point of view, the theory that autoimmunity/phlogosis may be considered as primary cause of MS seems to recall the Morgagni’s ideas, because the presence of autoimmune and inflammatory reactions in MS are surely unquestionable (albeit to different degree), but this by no means proposes that these reactions are the primary cause(s) of the disease. Furthermore, the fact that we can successfully reproduce these reactions in animal CNSs does not mean that they are the primary cause of the human disease; from a logical point of view, this way of reasoning is a paralogism, i.e., a faulty syllogism.

It would be also necessary to solve the riddle of which molecule of the myelinotrophic sequence Cbl→EGF→PrP^C^s decreases first in the MS CNS, eventually leading to the decrease in the others. Alternatively, the possibility that their decrease may be separated from each other, cannot be ruled out. On the basis of our knowledge of myelination and remyelination mechanisms, we can assert that any remyelination is doomed to failure in the MS CNS when these three molecules are lacking.

Even though cellular interactions between ODCs and the other myelinogenesis-involved CNS cells regulate the complex process of remyelination [58,63,65,155,479,480], ODCs themselves seem to be ultimately responsible for the failure of myelin repair in MS. Indeed, specific ODC defects (e.g., the inappropriate production of growth factors) may trigger and thereby contribute to demyelinating inflammatory/autoimmune reactions in MS [481]. From the angle of new MS therapies, it seems to be more appropriate to look at the myelinogenic ability of ODCs and the potential capacity of development and migration of NSCs [482]. Nevertheless, the matter is further complicated by the fact that: (a) a vicious circle could be set up in MS and be responsible, at least in part, for remyelination failure, because ODC abnormalities might bring about a lack of EGF and PDGF (especially in the terminal phases of the disease) [288]; this lack could, in turn, hamper the differentiation and proliferation of new OPC→ODC lineage cells; (b) MS remyelination is quantitatively erratic, as it is successful in some patients but not in others [1,2,3,4,5,48]; (c) MS remyelination of cerebral cortex occurs in patients regardless of disease duration [64]; and (d) demyelinated axons can rebuild their membranes, in some cases via changes in ion channel organization that support the restoration of impulse conduction after demyelination [483].

On the assumption that enhancing endogenous NSC proliferation and their differentiation towards OPC→ODC cell lineage by physiological molecules (e.g., some suitable growth factors) in MS lesions could lead to a successful remyelination therapy, some authors have argued that EGF could theoretically be a promising candidate for such therapy as a physiological enhancer of NSC mobilization and OPC→ODC lineage differentiation [342,484]. Therefore, it seems more profitable to continue the investigation of MS tissue samples (obtained by biopsies or autopsies), and to collect a set of focused information, which could lead us hopefully to uncover the answer to the problem of MS pathogenesis. In other words, before NSC transplantation we must harness the possibility of endogenous remyelination capacity of the adult CNS [85], in which adult SVZ NSCs contribute significantly to the regeneration of, and remyelination by, ODCs [127,482]. At the same time, we have to search for reducing the overexpression of the physiological inhibitors of the myelination cellular program [485].

Let me close by quoting a statement of the philosopher L. Anino Seneca (first century after Christus) taken from “Naturales Quaestiones” (“Natural questions”): “Veniet tempus quo ista quae nunc latent in lucem dies extrahat et longioris aevi diligentia” (the time will come in which the passing of time and the painstaking research of the future generations will shed new light upon the knowledge that is now obscure) (liber/book VII 25), although Marcel Proust wrote in “À la recherche du temps perdu”, La Prisonniere” (“In search of lost time”, “The Prisoner”) (1923):”L’inconnu de la vie des êtres est comme celui de la nature, que chaque découverte scientifique ne fait que reculer mais n’annule pas” (The unknown of the life of human beings is like the unknown of the nature, that each scientific discovery can only reduce but never abolish).

## Figures and Tables

**Table 1:** A list of some changes in the MS CNS that are inconsistent with an autoimmune-mediated pathogenesis of MS.

Change	References
Decreased PrP^C^ levels	[46,48]
Abnormalities in myelin structure	[88,89,90,91,92,93,94,95,96,97]
Changes in ODC transcriptome	[104,105,106,107,280]
Changes in acetylation and/or deacetylation levels of histones	[107,439]
Changes in ECM composition leading to a predominance of inhibitors of OPC differentiation and proliferation over promoters	[279,288,479,485]
Increased levels of inhibitors of differentiation and maturation inside the OPC→ODC cell lineage	[279,288,479,485]
Increased glutamate levels	[288]
Decreased levels of dome ODC- and myelino-trophic factors (mainly PDGF and EGF)	[288,357,359]
Opposite changes in the Cbl levels of SC and CSF	[357,359]
Changes in DNA methylation level	[361,362,363,436]
Reduced efficiency of mitochondrial respiratory chain	[411,412,416]
Decreased levels of some ‘’neurosteroids’’	[421,432,433]
Changes in miRNA profiles	[444,453,454,455]
Dysregulated neuronal Na^+^ channel expression	[483]
Increased GFAP levels	[486,487]
Abnormalities in myelin composition	[488]

Cbl = Cobalamin; CSF = Cerebrospinal fluid; ECM = Extracellular matrix; EGF = Epidermal growth factors; GFAP = Glial fibrillary acid protein; MS = Multiple sclerosis; ODC = Oligodendrocyte; OPC = Oligodendrocyte precursor cell; PDGF = Platelet-derived growth factor; PrP^C^ = Normal cellular prion protein; SC = Spinal cord.

## Data Availability

Not applicable.

## Abbreviations

Ab	Antibody
AST	Astrocyte
Cbl	Cobalamin
CNS	Central nervous system
CSF	Cerebrospinal fluid
EAE	Experimental allergic encephalomyelitis
ECM	Extracellular matrix
EGF	Epidermal growth factor
Erk	Extracellular signal-related kinase
GFAP	Glial fibrillary acidic protein
GS	Glutamine synthetase
HB	Heparin-binding
IGF	Insulin-like growth factor
MAG	Myelin associated glycoprotein
MBP	Myelin basic protein
miRNA	micro-RNA
MOG	Myelin oligodendrocyte-specific glycoprotein
mTORC	Mammalian target of rapamycin complex
NEU	Neuron
NF-κB	Nuclear factor kappa-light chain of B cell
NRG	Neuregulin
NSC	Neural stem cell
ODC	Oligodendrocyte
OPC	Oligodendrocyte precursor cell
PDGF	Platelet-derived growth factor
PLP	Proteolipid protein
PP	Primary progressive
PrP^C^	Normal cellular prion protein
PrP^SC^	Scrapie prion
RR	Relapsing and remitting
SC	Spinal cord
Shh	Sonic hedgehog
SP	Secondary progressive
SVZ	Subventricular zone
tHCYS	total Homocysteine
TNF	Tumour necrosis factor
